# Public health implications of the send-down movement: long-term effects on cognitive ability of rural older adults in China

**DOI:** 10.3389/fpubh.2025.1581826

**Published:** 2025-05-29

**Authors:** Guangchuan Zhao, Chen Su

**Affiliations:** ^1^School of Economics, Fudan University, Shanghai, China; ^2^School of Public Administration, Nanjing University of Finance and Economics, Nanjing, China; ^3^Institute of Social Security, Nanjing University of Finance and Economics, Nanjing, China

**Keywords:** cognitive ability, public health, send-down youths, education, China

## Abstract

**Objective:**

This study examines the long-term effects of the send-down movement on the cognitive ability of rural older adults in China, focusing on how early-life exposure to human capital interventions shapes late-life cognitive trajectories.

**Methods:**

Leveraging four waves of the China Health and Retirement Longitudinal Study (CHARLS, 2011–2018), we employ a cohort difference-in-differences (cohort DID) design to compare cognitive outcomes between rural residents exposed to send-down youths (SDYs) during childhood and non-exposed cohorts. Mechanisms are analyzed through a multi-mediation framework integrating educational attainment, non-agricultural work, social engagement, and fertility behaviors.

**Results:**

The analysis demonstrates that exposure to SDYs significantly enhanced cognitive ability among rural older adults, resulting in a 0.857-point increase in cognitive ability score, a 4.33 percentage-point reduction in cognitive decline, and a 6.76 percentage-point decrease in cognitive impairment incidence. Mechanism analysis reveals that exposure to SDYs primarily influenced late-life cognitive ability through four pathways: improving rural children's educational attainment, increasing their probability of obtaining non-agricultural work, enhancing social engagement, and reducing fertility rates.

**Conclusion:**

The send-down movement positively influenced the cognitive health of rural older adults, underscoring the enduring impact of childhood access to educational resources on cognitive ability throughout the life course. Policy initiatives integrating early-life education with adult opportunity structures could yield compounded cognitive dividends, particularly in resource-limited rural settings.

## 1 Introduction

Amid accelerating global population aging, cognitive health has become a central public health priority (1). Cognitive impairments, including Alzheimer's disease and related dementias, represent the leading causes of disability, diminished quality of life, and socioeconomic burden among older adults worldwide (2). China, experiencing one of the most rapid aging transitions, accounts for a quarter of global dementia cases (3). In 2020, ~10 million Chinese lived with Alzheimer's disease, a figure projected to exceed 20 million by 2050 (4). Cognitive decline not only undermines individuals' independence but also intensifies familial caregiving burdens and strains healthcare systems, posing critical challenges to sustainable aging policies.

Life-course epidemiology reveals that cognitive aging is not solely driven by biological processes but shaped by dynamic interactions between early-life experiences and socioenvironmental contexts. Childhood access to education, socioeconomic resources, and knowledge acquisition critically influences late-life cognitive ability by shaping cognitive reserve and neural plasticity (5). This mechanism is particularly pronounced in resource-limited rural areas, where early educational deprivation may permanently impair resilience against neurodegenerative aging (6). Against this backdrop, China's send-down movement (1968–1978)—a historical policy relocating 17 million urban youths to rural areas—provides a unique natural experiment to investigate the lifelong health impacts of early-life human capital interventions.

As one of the largest urban-to-rural population mobility in modern history, this movement inadvertently transformed rural educational ecosystems and social networks. Economic studies demonstrate that SDYs, acting as cultural intermediaries, significantly increased educational attainment among rural children—particularly girls—and reduced regional educational inequalities (7). However, existing literature predominantly focuses on outcomes like educational gains and female empowerment (8), neglecting critical public health questions: (1) How does childhood exposure to knowledge diffusion attenuate cognitive decline decades later? (2) Through what mechanisms do educational improvements interact with adult occupational choices, social engagement, and fertility behaviors to shape cognitive trajectories? Addressing these gaps holds vital implications for designing cost-effective cognitive health strategies in aging societies.

This study bridges these gaps by leveraging four waves of the CHARLS data (2011–2018) to assess the send-down movement's enduring cognitive impacts. Employing a cohort DID design, we find that rural residents exposed to SDYs during childhood exhibited a 0.857-point increase in cognitive scores, a 6.76-percentage-point reduction in cognitive impairment incidence, and a 4.33-percentage-point slower cognitive decline rate. Mechanism analyses identify four mediating pathways: (1) education-driven cognitive reserve accumulation, (2) cognitive stimulation from non-agricultural work, (3) cognitive activation through expanded social networks, and (4) mitigation of family resource dilution via reduced fertility. Notably, education amplifies the benefits of non-agricultural work and social engagement, underscoring its role as a “leverage point” that magnifies the health returns of socioeconomic opportunities.

The main contributions of this paper include: first, we empirically examine the long-term impact of exposure to SDYs on the cognitive ability of older adults in rural China using a cohort DID model. Second, we delve into the mechanisms underlying these effects, proposing that the movement influences cognition through four pathways: enhancing educational attainment, increasing opportunities for non-agricultural work, boosting social engagement, and reducing fertility rates. Additionally, we innovatively identify the role of education in the other three pathways, highlighting its critical importance for cognitive capacity.

The structure of the paper is as follows: Section 2 details the institutional background of the send-down movement, including a brief overview of its development process, characteristics of SDYs involved, and impacts on rural areas; Section 3 presents theoretical analysis and research hypotheses; Section 4 outlines the research design, covering data sources, variable definitions, and empirical models; Section 5 provides empirical results analysis, including parallel trend tests for cohort DID, baseline regression results, and mechanism analysis; Section 6 includes robustness checks; and Section 7 concludes.

## 2 Institutional background: the send-down movement in China

### 2.1 A brief history

The send-down movement, spanning nearly three decades since the 1950s, was a large-scale state-led social mobilization policy aimed at resolving structural urban-rural disparities. Its institutional framework evolved from grassroots experimentation to compulsory state enforcement. Initiated in 1955 when Beijing youth Yang Hua and others formed the first voluntary reclamation team—marking the movement's inception—it gained formal policy recognition with Mao Zedong's proclamation “*The countryside is a broad arena where one can make significant contributions*,” articulated in his annotations to *The Upsurge of Socialism in China's Rural Areas*. In 1962, facing food shortages induced by the “3-year natural disaster,” the central government for the first time systematically mobilized urban youths to resettle in the countryside, but before 1966, this was mainly voluntary, with a cumulative total of 1.29 million people (9). The movement escalated dramatically in December 1968 under Mao's directive “*Urban youths must go to the countryside to be re-educated by poor and lower-middle peasants*,” which, against the backdrop of the Cultural Revolution, mandated the relocation of 17.7 million youths by 1978. The peak occurred during 1967–1969, with 4.7 million “Old Three Graduates” (graduates from 1966 to 1968) forcibly resettled.

**Figure 1:**
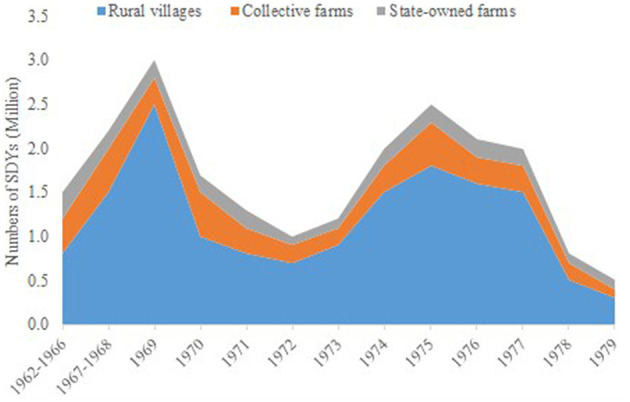
Numbers of SDYs by resettlement, 1962–1979. This figure is sourced from the work of Gu (11).

Resettlement took three primary forms: insertion into rural villages (*chadui*)—accounting for 73% (~12.8 million)—collective farms, and state-owned farms (Figure 1). Despite its ideological framing as a “re-education” initiative, most urban youths engaged in non-agricultural technical roles (e.g., teaching, healthcare, or agricultural innovation), bridging urban-rural knowledge gaps. By 1978, widespread protests (notably the 1978 “Return-to-City Wave” in Yunnan) compelled the central government to terminate the policy in 1980, with 95% of youths returning to cities while 5% remained in rural areas due to marriage or non-agricultural job placements (10). This concluded this social engineering project, which interwove political mobilization, urban employment alleviation, and rural modernization objectives.

### 2.2 Flows of SDYs

The SDYs primarily originated from core cities with high urbanization rates, notably the major municipalities of Beijing, Shanghai, and Tianjin (11). As detailed in Table 1, these three cities dispatched 636.3 thousand, 1252.2 thousand, and 465.1 thousand SDYs respectively between 1962 and 1979, with substantial proportions assigned to cross-provincial destinations (39.6%, 57.5%, and 58.4%, respectively). Collectively, these municipalities contributed 1,243.5 thousand cross-provincial migrants—representing 87.3% of the total national interprovincial relocation (1,423,800). Geographically, 93% of SDYs were resettled within their home provinces. For instance, Hebei Province received 510.5 thousand SDYs, 74% of whom originated locally. However, border regions—driven by labor demands and strategic planning—absorbed the majority of cross-provincial flows. Heilongjiang, Inner Mongolia, Yunnan, and Xinjiang collectively received 754.1 thousand interprovincial youths (53% of the national total).

**Table 1:** Total number of sent and received SDYs in each province, 1962–1979.

**Provinces**	**SDYs received (Thousand)**			**SDYs sent (Thousand)**		
	**Total**	**Inside**	**Outside**	**Total**	**Inside**	**Outside**
Beijing	384.2	384.2	0.0	636.3	384.2	252.1
Tianjin	193.6	193.6	0.0	465.1	193.6	271.5
Hebei	510.5	377.8	132.7	384.4	377.8	6.6
Shanxi	312.9	264.3	48.6	264.3	264.3	0.0
Inner Mongolia	299.3	193.8	105.5	193.8	193.8	0.0
Liaoning	2,018.0	2,013.4	4.6	2,013.4	2,013.4	0.0
Jilin	1,052.6	991.4	61.2	991.4	991.4	0.0
Heilongjiang	1,922.2	1,519.2	403.0	1,519.2	1,519.2	0.0
Shanghai	532.3	532.3	0.0	1,252.2	532.3	719.9
Jiangsu	861.2	810.2	51.0	828.4	810.2	18.2
Zhejiang	595.9	563.9	32.0	646.2	563.9	82.3
Anhui	725.5	576.5	149.0	576.5	576.5	0.0
Fujian	372.3	372.3	0.0	372.3	372.3	0.0
Jiangxi	622.5	504.5	118.0	504.5	504.5	0.0
Shandong	492.7	492.7	0.0	512.9	492.7	20.2
Henan	673.0	673.0	0.0	673.0	673.0	0.0
Hubei	878.6	878.6	0.0	886.6	878.6	8.0
Hunan	635.8	635.8	0.0	635.8	635.8	0.0
Guangdong	973.2	973.2	0.0	973.2	973.2	0.0
Guangxi	434.8	434.8	0.0	434.8	434.8	0.0
Sichuan	1,427.4	1,427.4	0.0	1,472.4	1,427.4	45.0
Guizhou	224.1	213.5	10.6	213.5	213.5	0.0
Yunnan	339.1	232.5	106.6	232.5	232.5	0.0
Tibet	3.4	3.4	0.0	3.4	3.4	0.0
Shaanxi	490.3	463.1	27.2	463.1	463.1	0.0
Gansu	264.3	245.2	19.1	245.2	245.2	0.0
Qinghai	51.0	43.6	7.4	43.6	43.6	0.0
Ningxia	57.5	49.2	8.3	49.2	49.2	0.0
Xinjiang	416.6	277.6	139.0	277.6	277.6	0.0
Total	1,7764.8	1,6341.0	1,423.8	1,7764.8	1,6341.0	1,423.8

This table is obtained from Table 1 of Chen et al. (7), originally sourced from the work of Gu (11).

This distribution underscores the policy's prioritization of geographic proximity and economic viability. Most SDYs were allocated to nearby rural areas, while border regions aggregated cross-provincial labor through structured entities such as the Heilongjiang Production and Construction Corps (which absorbed 390 thousand youths). Crucially, this spatial strategy prioritized economic development and national defense imperatives over improving rural health, highlighting the movement's role as an exogenous policy intervention.

### 2.3 The influence on rural china

The send-down movement represents a significant social phenomenon in Chinese history with far-reaching implications. With the assistance of SDYs, basic education in many rural areas improved significantly, particularly through the supplementation of the teaching workforce. Those SDYs filled critical educational gaps, introducing modern scientific knowledge and urban culture to rural children, thereby broadening their horizons and enriching their understanding of the outside world. This interaction not only enhanced the educational quality of rural children but also subtly promoted the modernization of rural culture. Even after the conclusion of the movement and the subsequent return of SDYs to urban areas, their ties with rural communities remained intact. In fact, many SDYs maintained their connections to rural areas during China's reform and opening-up period, contributing through investments in local businesses, donations to education, and participation in charitable activities. They helped the next generation gain exposure to urbanization and modernization, motivating them to pursue higher education and migrate to cities, thus serving as a bridge between urban and rural areas.

For this study, the send-down movement, as a state-enforced social experiment of relatively short duration, offers a unique perspective. The movement was a policy-driven initiative, with most participants involuntarily involved, effectively minimizing selection bias and resulting in a relatively homogeneous group. The clear timeline of the movement enables the construction of accurate empirical models to explore its potential impact on the cognitive ability of rural children in later life, thereby reducing interference from other confounding factors. Moreover, the enduring connection between SDYs and rural areas after the movement's conclusion provides rich social context and empirical data for analyzing the underlying mechanisms. This supports the theoretical research and policy recommendations presented in this paper.

## 3 Theoretical analysis and research hypotheses

Cognitive ability, defined as an individual's capacity to process information, solve problems, acquire knowledge, and apply learned skills, forms the foundation for decision-making. With aging, cognitive trajectories reflect dynamic interactions influenced by biological and experiential forces (12), as articulated by the dual-process theory of intellectual development (13). This theory distinguishes between crystallized intelligence—knowledge and skills accumulated through education and experience (e.g., vocabulary retention), which remains stable or improves with age—and fluid intelligence, the biologically rooted ability for abstract reasoning and novel problem-solving that declines with aging (14). These dynamics highlight that cognitive decline is not solely determined by biological factors but is profoundly shaped by early-life experiences, particularly childhood environments, and educational exposure.

Childhood socioeconomic conditions exert enduring effects on late-life cognition. For instance, Modrek et al. (15) demonstrated that male children in U.S. regions with intensive work relief programs during the Roosevelt New Deal era exhibited higher cognitive scores in later life. Similarly, Banks and Mazzonna (16) found that the 1947 UK compulsory education reform, which raised the minimum school-leaving age from 14 to 15, improved older adults' cognitive outcomes. In China, children vaccinated before age 15 gained ~1 additional year of schooling, correlating with better cognitive performance in old age (17). These findings underscore the lifelong imprint of early-life socioeconomic conditions.

Education, as a critical component of human capital, plays a pivotal role in shaping cognitive trajectories. Studies reveal that education not only directly enhances cognitive abilities but also confers lifelong protective effects against age-related decline (18). Higher educational attainment, particularly early education, delays cognitive aging and mitigates dementia risk (19, 20), thereby reducing public health burdens associated with neurodegenerative diseases (21). Improving early educational access and quality thus represents a strategic intervention to combat late-life cognitive impairment (22).

Within the context of China's sent-down movement, we propose the first two hypotheses:

**Hypothesis 1:** Exposure to SDYs contributes to improving the cognitive ability of rural older adults, slowing down their cognitive decline, and reducing the occurrence of cognitive impairments.
**Hypothesis 2:** Exposure to SDYs affects the cognitive ability of rural older adults by increasing educational opportunities in rural areas.

Elevated rural education levels often drive labor migration to urban areas, where occupations involve greater complexity compared to rural farming. Such cognitively stimulating environments may delay age-related decline (23). Rural migrants engaged in urban non-agricultural work also exhibit better mental health (24). Urbanization further facilitates broader social networks, which protect against cognitive decline (25), whereas social isolation correlates with poorer cognition (26). Robust social networks also mitigate mental health strains from rural-urban migration (27).

Additionally, the urban fertility decline in China—partly driven by job insecurity fears among employees (28)—may intersect with the movement's influences. Returnees maintained rural ties, potentially shaping migrants' fertility behaviors through urban norms. Studies indicate that having four or more children (vs. two) negatively correlates with immediate and delayed word recall (29), and three or more children (vs. two) impair late-life cognition (30). Based on these mechanisms, the following hypothesis is proposed:

**Hypothesis 3**: Exposure to SDYs increases the likelihood of rural people engaging in non-agricultural work, expands their social networks, and reduces the number of children they have, thereby improving their cognitive ability in later life, with education potentially playing an indirect role in this process.

**Figure 2:**
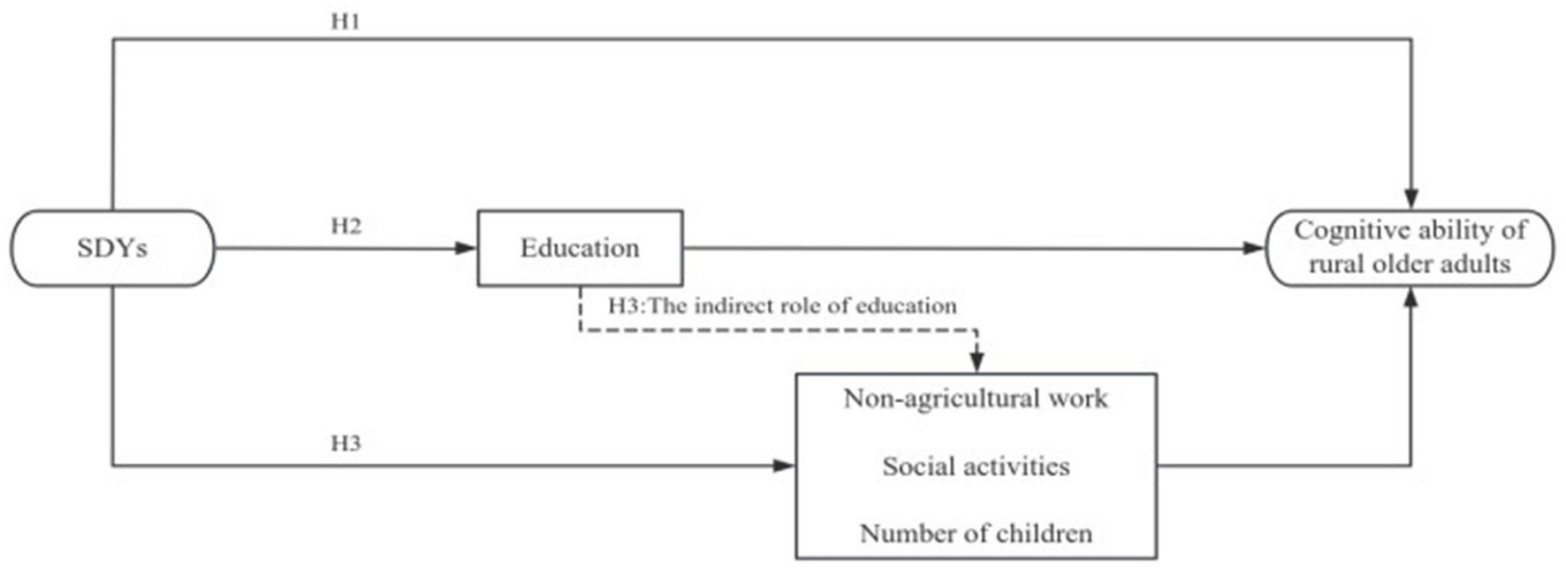
Theoretical framework of the send-down movement's impact on cognitive ability.

These hypotheses, integrated into the conceptual framework illustrated in Figure 2, posit that the movement shaped the cognitive ability of rural older adults through human capital accumulation and behavioral adaptations. This multichannel perspective advances the understanding of how historical policy interventions interact with life-course mechanisms to influence cognitive aging, offering actionable insights for public health strategies targeting dementia prevention.

## 4 Research design

### 4.1 Data source

The data for this study were drawn from two primary sources. First, information on the scale and density of SDYs was obtained from the county gazetteer dataset compiled by Chen et al. (7), which aggregates historical records from over 3,500 county-level chronicles to reconstruct standardized metrics for 2,868 counties. For detailed methodologies on dataset construction and validation, refer to Chen et al. (7).

Second, this study leverages individual-level data from the CHARLS, a nationally representative longitudinal survey focusing on individuals aged 45 and older, conducted by Peking University. The CHARLS employs multi-stage stratified probability sampling and provides comprehensive socioeconomic, demographic, and health-related variables collected through biennial waves since 2011. The CHARLS sample is drawn from 150 counties and 450 villages, involving more than 10,000 households, primarily including urban and rural residents aged 45 and above. As of now, the CHARLS data has been updated to 2020. However, due to the COVID-19 pandemic in 2020, the health status of the sample may have been affected. Therefore, the data selected for this study primarily comes from four waves of tracking survey data conducted between 2011 and 2018.

The sample selection was conducted as follows: first, observations from Beijing, Shanghai, and Tianjin—the primary sources of SDYs—were excluded to address spatial selection bias. Second, we retained only rural-born residents with agricultural hukou status who continued to reside in their birth counties, thereby controlling for confounding effects of urbanization and migration. Third, we restricted the birth cohort to 1946–1969 to align with the policy period (the 1950s−1980s) and establish temporally coherent control (pre-movement) and treatment (movement-affected) groups. Fourth, observations with missing values for key outcome variables were discarded. After these steps, 28,675 valid observations were retained for final analysis.

### 4.2 Variable definitions

#### 4.2.1 Measures of exposure to SDYs

**Figure 3:**
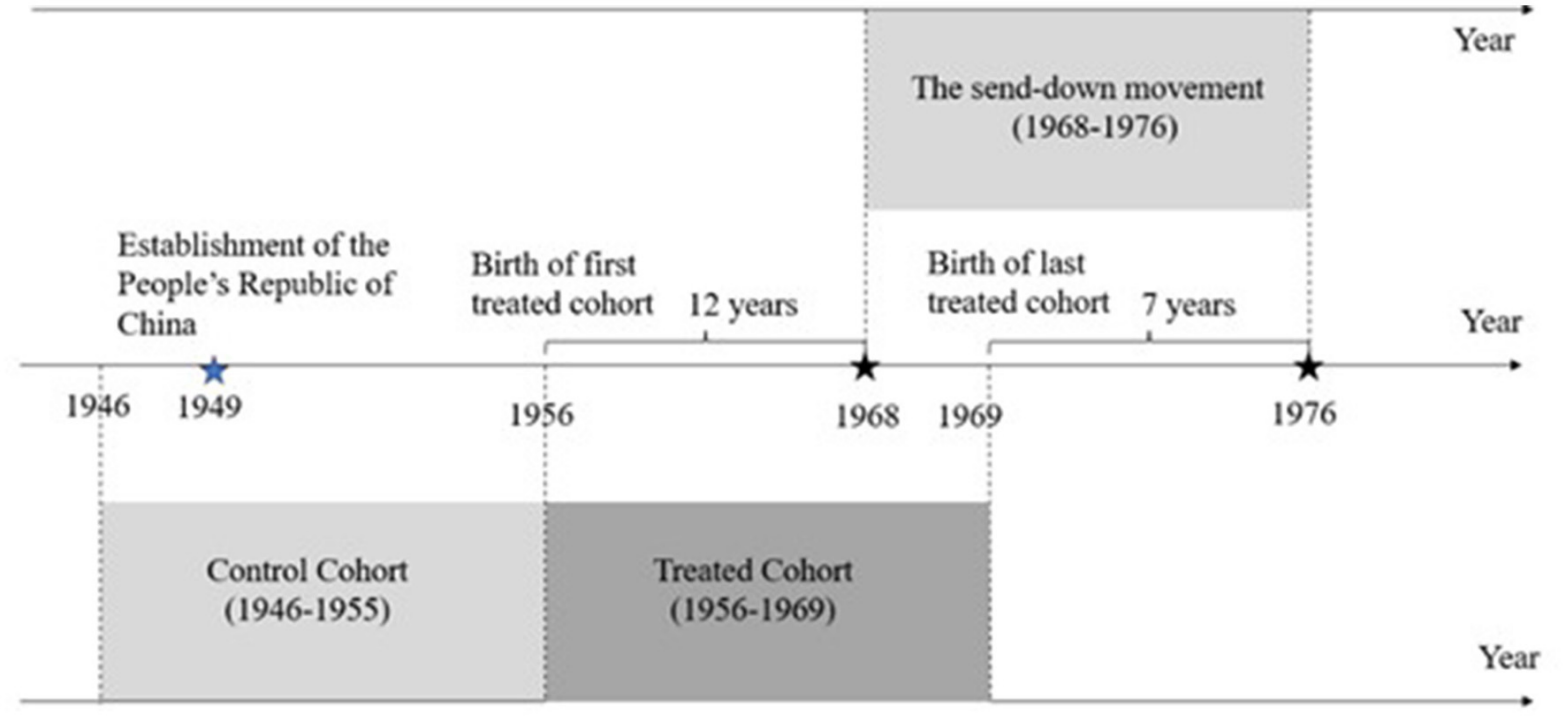
Timeline of the send-down movement. This figure is obtained from Figure A6 of Liu et al. (8). This figure presents the timeline of the send-down movement and other main social events relevant to our study after the establishment of the People's Republic of China.

To quantify the effect of exposure to SDYs on cognitive health, we defined two critical variables: the local density of SDYs at the city level and the age of individuals during the movement. Drawing on Liu et al. (8), we constructed a treatment group based on individual birth cohorts and the period of the movement under the DID framework. As mentioned earlier, SDYs were mainly engaged in elementary school teaching, therefore, the treatment group comprised individuals born between 1956 and 1969, who were attending primary school during the movement. Specifically, individuals born in 1956 entered Grade 6 in the initial year of the movement (1968), while those born in 1968 completed Grade 1 in the final year (1976). Individuals born in other years are no longer affected by the movement. A schematic of the treatment group construction process is given in Figure 3. Another dimension of variation in the DID framework comes from the local density of SDYs at the city level, measured as the total number of SDYs during 1968–1976 divided by the 1964 pre-movement population across all counties (7). The common interpretation is that the higher the density of SDYs, the more rural children are affected. After sample selection and data cleaning, the final CHARLS dataset contains SDY density data for 119 cities, with the average SDY density for the entire sample being 1.8%.

#### 4.2.2 Measures of cognitive ability

The dependent variable in this study is cognitive ability which includes three aspects: cognitive ability level, cognitive decline, and cognitive impairment. First, cognitive ability level is measured using the total score of the Mini-Mental State Examination (MMSE) (31), with a maximum score of 30 points. In the CHARLS surveys from 2011 to 2018, the MMSE consists of four parts: word memory, calculation ability, date, and drawing. In the word memory section, the interviewer provides ten words for the respondent to repeat in any order, and after 10 min, the respondent is asked to recall the words. One point is awarded for each correctly recalled word, with a maximum of 20 points. In the calculation section, the respondent needs to start from 100 and subtract 7 repeatedly five times. One point is awarded for each correct answer, with a maximum of 5 points. In the data section, the respondent must answer four questions: the current year, the current month, today's date, and the day of the week. One point is awarded for each correct answer, with a maximum of 4 points. In the drawing section, the respondent is asked to replicate a given figure, and one point is awarded for correct replication, with a maximum of 1 point. Second, cognitive decline in this study is defined as a relative decrease in memory ability, with the difference in word memory score between the current and previous survey being greater than or equal to 20% of the previous total score, as per Mazzonna and Peracchi (32). This measure is used to assess cognitive decline in rural older adults. Third, following the criteria set by Monroe and Carter (33), cognitive impairment in this study is defined as a MMSE score of 17 or below.

#### 4.2.3 Control variables

The control variables in this study mainly include individual-level and family-level factors (see Table 2). The individual-level control variables consist of gender, ethnicity, marital status, place of residence, employment status, activities of daily living (ADLs), chronic disease status, drinking, smoking, and public health insurance coverage. The family-level control variables include whether the individual lives with children and the household's per capita annual income (log-transformed). Since age is already controlled when setting up the treatment and control groups, it is not included as a control variable at the individual level.

**Table 2:** Descriptive statistics.

**Variables**	**Control group (1946–1955)**		**Treatment group (1956–1969)**	
	**Mean**	**Std. Dev**.	**Mean**	**Std. Dev**.
MMSE score	13.800	4.571	15.670	4.524
Cognitive decline	0.376	0.485	0.292	0.454
Cognitive impairment	0.785	0.411	0.638	0.480
Local density of SDYs	0.018	0.016	0.018	0.017
Gender (Male = 1)	0.545	0.498	0.496	0.500
Ethnicity (Han = 1)	0.929	0.257	0.920	0.271
Marital status	0.847	0.360	0.870	0.336
Residence (Urban = 1)	0.241	0.428	0.258	0.438
Employment status	0.745	0.436	0.844	0.363
ADLs	0.003	0.053	0.001	0.032
Chronic diseases (Yes = 1)	0.781	0.414	0.682	0.466
Drinking	0.494	0.500	0.456	0.498
Smoking	0.493	0.500	0.422	0.494
Public health insurance	0.960	0.196	0.958	0.200
Living with children	0.484	0.500	0.620	0.485
Household income per capita	7.384	2.739	7.307	3.703

### 4.3 Empirical model

We use a cohort DID model to identify the impact of the sent-down movement on the cognitive ability of rural older adults, considering two main sources of variation. First, the number of SDYs received by different cities during the movement varied. Second, even within the same city, the effect of the movement on individuals from the same birth cohort is influenced by the overlap of the timing when different cohorts started primary school. Therefore, based on the study by Chen et al. (7), we estimate the following cohort DID model:


(1) $$\begin{array}{l} Cognition_{i\text{g}ct}=\beta_{0}+\beta_{1}SDY_{c}\times I\left(1956\leq g\leq 1969\right)+\beta_{2}X_{i\text{g}ct}\\ \;+\lambda_{c}+\lambda_{g}+\lambda_{t}+\varepsilon_{i\text{g}ct} \end{array}$$

where *Cognitio*_*n*_*igc*_*t*_ represents the cognitive ability in year *t* for individual *i* born in city *c* in cohort year *g*. *SDY*_*c*_ is the local density of SDYs in city *c* during the movement. The indicator function *I*(1956 ≤ *g* ≤ 1969) assigns treatment status such that individuals born between 1956 and 1969 are classified as the treatment group, while those born from 1945 to 1955 serve as the control group. A schematic representation of this cohort categorization is provided in Figure 3. The coefficient β_1_associated with the interaction term constitutes the focal parameter of interest, identifying the causal effect of the movement within the DID framework. _*X*_*igc*_*t*_ includes the individual and family-level control variables for cohort *g* born in city *c* in year *t*; λ_*c*_, λ_*g*_ and λ_*t*_ represent city fixed effects, cohort fixed effects, and year fixed effects, respectively; is the error term, and the standard errors are clustered at the city level.

## 5 Empirical results

### 5.1 Parallel trend test

**Figure 4:**
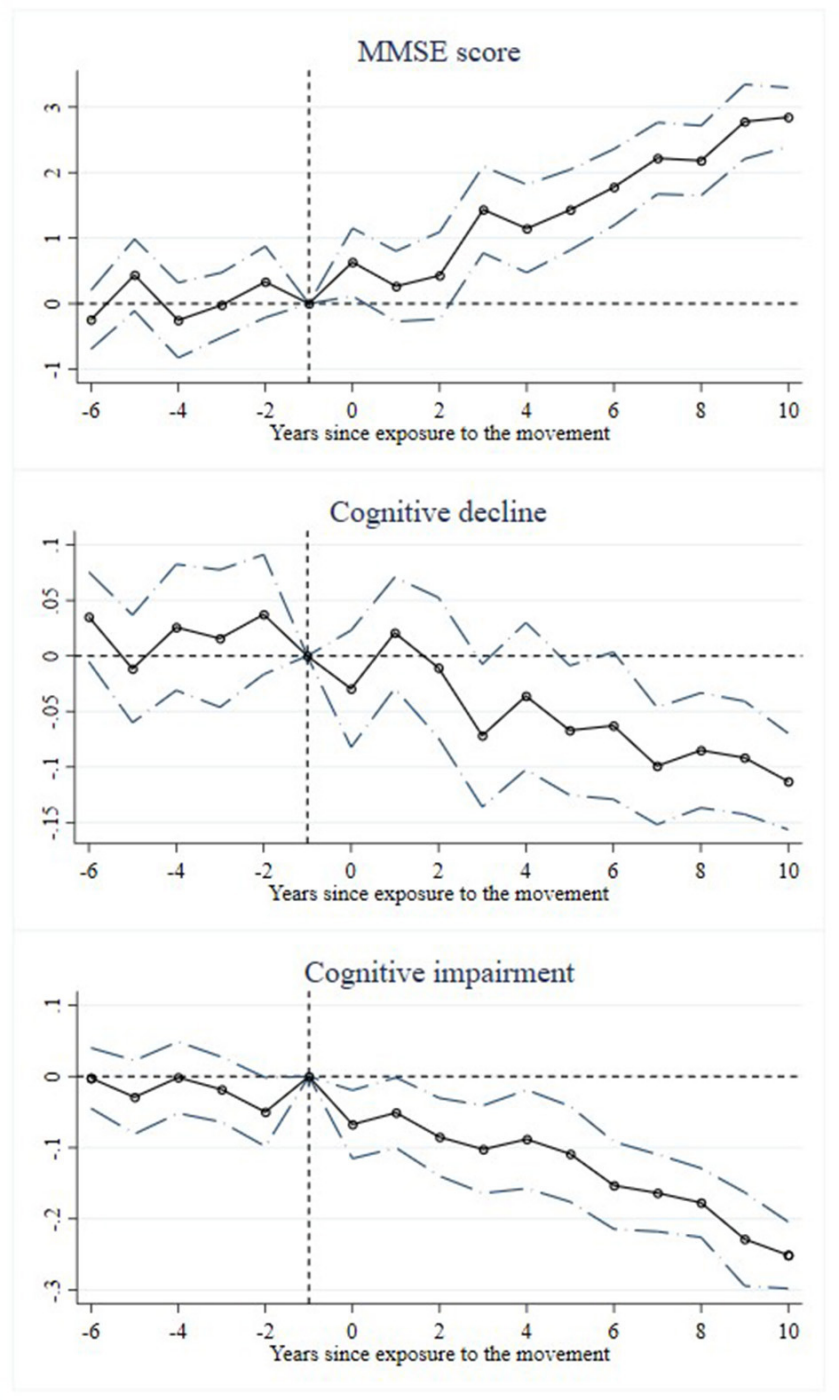
Event study on the send-down movement's effect on cognitive ability.

This study adopts the cohort DID method to identify the causal effect of exposure to SDYs on the cognitive ability of rural older adults. A key assumption in this method is the parallel trend hypothesis, meaning that before the movement, the cognitive ability trends between the treatment group and the control group should have been parallel. To test this, we set the born year (1955) before the movement as the baseline year and further refine the baseline regression model as follows:


(2) $$\begin{array}{l} Cognition_{igct}=\beta_{0}+\overset{1969}{\underset{\gamma =1946,\gamma \neq 1955}{\sum }}\beta_{\gamma }SDY_{c}\times I\left(g=\gamma \right)\\ \;+\beta_{2}X_{igct}+\lambda_{c}+\lambda_{g}+\lambda_{t}+\varepsilon_{igct} \end{array}$$

where *I*(*g* = γ) denotes that cohort *g* was born in year γ. Other variables are specified the same as Equation 1. Figure 4 presents the coefficients estimated based on Equation 2, with 95% confidence intervals. As shown in Figure 4, before the movement in 1956, there was no significant difference in cognitive ability between the treatment and control groups. However, after the movement, the treatment group showed a significant improvement in cognitive ability, a slower decline in cognition, and a notable reduction in the incidence of cognitive impairment. This result suggests that the impact of the movement on the cognitive ability of rural older adults is statistically significant, supporting Hypothesis 1 of this study.

### 5.2 Estimation results

Table 3 presents the effects of the movement on the cognitive ability of rural older adults across three dimensions: cognitive ability level measured by MMSE score, cognitive decline, and cognitive impairment. Overall, the results demonstrate that exposure to SDYs significantly improved the cognitive ability of rural older adults. Specifically, Column (1) reveals that rural older adults exposed to SDYs experienced a statistically significant enhancement in cognitive ability, with a coefficient of 47.630. Given that the average SDY density is 1.8% (i.e., 18 out of every 1,000 residents were SDYs), this implies that exposure to SDYs increased the MMSE scores of rural older adults by 0.857 points (47.630 × 1.8%). Columns (2) and (3) present the effects of exposure to SDYs on cognitive decline and cognitive impairment, respectively. The results show that exposure to SDYs reduced the rate of cognitive decline by 4.33 percentage points (2.406 × 1.8%) and lowered the probability of cognitive impairment by 6.76 percentage points (3.753 × 1.8%). These empirical findings provide strong support for Hypothesis 1, indicating that exposure to SDYs not only had a protective effect on the cognitive ability of rural older adults but may also have contributed to their cognitive improvement.

**Table 3:** The effect of exposure to SDYs on the cognitive ability of the older adults in rural China.

**Variables**	**MMSE score**	**Cognitive decline**	**Cognitive impairment**
	**(1)**	**(2)**	**(3)**
local density of received SDYs × affected cohorts (1956–1969)	47.630^***^	−2.406^***^	−3.753^***^
	(10.741)	(0.532)	(0.936)
Controls	Yes	Yes	Yes
Cohort FE	Yes	Yes	Yes
City FE	Yes	Yes	Yes
Year FE	Yes	Yes	Yes
Observations	14,671	9,636	14,671
*R* ^2^	0.110	0.035	0.070

Standard errors are clustered at the city level. Local density of received SDYs is defined as the number of received SDYs in one city during 1968–1977 divided by the total population of all counties in the city in 1964. ^***^, ^**^ and ^*^ denote significance at 1%, 5% and 10% level, respectively.

### 5.3 Mechanism analysis

Based on the literature review, exposure to SDYs influences cognitive ability through four primary mechanisms: education, non-agricultural work, social engagement, and changes in fertility. This section empirically examines these mechanisms.

First, using education-related variables from the CHARLS dataset, we constructed three indicators: years of education, graduation from junior high school, and graduation from senior high school. Years of education were coded as follows: below primary school as 0 years, primary school graduation as 6 years, junior high school graduation as 9 years, high school or technical school graduation as 12 years, college graduation as 15 years, a bachelor's degree as 16 years, and a master's degree or higher as 19 years. Graduation from junior high school and graduation from senior high school were treated as binary variables. As shown in Table 4, exposure to SDYs significantly increased the educational attainment of rural children, leading to an average increase of 1.17 years of schooling (65.010 × 1.8%). It also raised the graduation from junior high school rate by 12.51 percentage points (6.952 × 1.8%) and the graduation from senior high school rate by 3.66 percentage points (2.031 × 1.8%), with all results statistically significant at the 1% level. These findings suggest that exposure to SDYs had a substantial and positive impact on the educational outcomes of rural children.

**Table 4:** The effect of exposure to SDYs on the cognitive ability of rural older adults: the educational mechanism.

**Variables**	**Years of education**	**Graduate from junior high school**	**Graduate from senior high school**
	**(1)**	**(2)**	**(3)**
Local density of received SDYs × affected cohorts (1956–1969)	65.010^***^	6.952^***^	2.031^***^
	(13.920)	(1.605)	(0.508)
Controls	Yes	Yes	Yes
Cohort FE	Yes	Yes	Yes
City FE	Yes	Yes	Yes
Year FE	Yes	Yes	Yes
Observations	14,671	14,671	14,671
*R* ^2^	0.242	0.171	0.071

Standard errors are clustered at the city level. ^***^, ^**^ and ^*^ denote significance at 1%, 5% and 10% level, respectively.

Second, we defined non-agricultural work as whether an individual was engaged in non-agricultural work, coded as 1 for “yes” and 0 for “no.” Social engagement was coded as 1 if an individual participates in at least one of the following six activities: interacting with friends, playing mahjong or board games, engaging in sports or clubs, joining community organizations, volunteering or charity work, or attending educational/training courses. High fertility was operationalized as having three or more children (1 for yes, 0 otherwise). Table 5 reports the effects of exposure to SDYs on these three mediating variables. Columns (2), (4), and (6) introduce controls for educational attainment, whereas Columns (1), (3), and (5) present baseline estimates. This adjustment addresses the possibility that part of the SDYs exposure's impact on these mediators operates indirectly through improved education.

**Table 5:** The effect of exposure to SDYs on the cognitive ability of rural older adults: the other mechanism.

**Variables**	**Non-agricultural work**		**Social engagement**		**High fertility**	
	**(1)**	**(2)**	**(3)**	**(4)**	**(5)**	**(6)**
Local density of received SDYs × affected cohorts (1956–1969)	3.050^***^	2.580^***^	2.298^***^	1.886^***^	−8.585^***^	−8.241^***^
	(0.936)	(0.863)	(0.384)	(0.376)	(1.316)	(1.270)
Years of education		0.007^***^		0.006^***^		−0.005^***^
		(0.001)		(0.001)		(0.001)
Controls Cohort FE	Yes	Yes	Yes	0.330^***^	0.610^***^	0.620^***^
	Yes	Yes	Yes	(0.0456)	(0.0492)	(0.0488)
City FE	Yes	Yes	Yes	Yes	Yes	Yes
Year FE	Yes	Yes	Yes	Yes	Yes	Yes
Observations	14,671	14,671	14,670	14,670	14,671	14,671
*R* ^2^	0.239	0.244	0.058	0.061	0.214	0.215

Standard errors are clustered at the city level. ^***^, ^**^ and ^*^ denote significance at 1%, 5% and 10% level, respectively.

The results in Table 5 indicate that controlling for years of education reduces the coefficient estimates of the SDY exposure's effects on all three mediators. This suggests that omitting education would overstate the mediating roles of non-agricultural work, social engagement, and high fertility. Specifically, after accounting for education, exposure to SDYs increased the probability of non-agricultural employment by 4.6 percentage points (2.580 × 1.8%), raised the likelihood of social engagement by 3.4 percentage points (1.886 × 1.8%), and decreased the probability of high fertility by 14.83 percentage points (8.241 × 1.8%). These findings corroborate the multifaceted and enduring impact of exposure to SDYs on rural residents' livelihoods, extending even into late-life cognitive outcomes.

## 6 Robustness checks

This paper conducts multiple robustness checks on key variable settings to ensure the reliability of the results. First, to mitigate potential differences in cognitive ability caused by age discrepancies between the treatment and control groups, the bandwidth between the two groups is narrowed. Specifically, Column (1) of Table 6 sets the bandwidth to 8 years (adjusting the control group to 1948–1955 and the treatment group to 1956–1963), while Column (2) sets the bandwidth to 5 years (adjusting the control group to 1951–1955 and the treatment group to 1955–1960). Under these two settings, the results remain statistically significant and consistent.

**Table 6:** Robustness check: changing the sample.

**Variables**	**MMSE score**	**Cognitive decline**	**Cognitive impairment**	**MMSE score**	**Cognitive decline**	**Cognitive impairment**
	**Treated (1956–1963) VS. Control (1948–1955)**			**Treated (1956–1960) VS. Control (1951–1955)**		
	**(1)**	**(2)**	**(3)**	**(4)**	**(5)**	**(6)**
Local density of received SDYs × affected cohorts	32.350^***^	−1.992^***^	−2.413^***^	11.770^***^	−1.325^***^	−1.287^**^
	(8.414)	(0.531)	(0.791)	(4.486)	(0.497)	(0.565)
Controls	Yes	Yes	Yes	Yes	Yes	Yes
Cohort FE	Yes	Yes	Yes	Yes	Yes	Yes
City FE	Yes	Yes	Yes	Yes	Yes	Yes
Year FE	Yes	Yes	Yes	Yes	Yes	Yes
Observations	10,737	6,981	10,737	6,585	4,233	6,585
*R* ^2^	0.102	0.036	0.061	0.103	0.047	0.059

Standard errors are clustered at the city level. ^***^, ^**^ and ^*^ denote significance at 1%, 5% and 10% level, respectively.

Second, the density of SDYs varied significantly across counties within the same city. In our baseline regression, the density of SDYs was measured at the city level by dividing the total number of SDYs across all counties within a city during 1968–1977 by the city's aggregate population in 1964. This approach could lead to measurement deviations: counties with inherently low SDYs densities might be misclassified as having high values, and vice versa. To address this potential bias, we reconstructed the SDYs density variable. First, we computed the density of SDYs at the county level and then assigned the median, maximum, and minimum values of these county-level densities across all counties within a city to individuals residing in that city, generating alternative density measures. Subsequently, we conducted separate regressions using each newly constructed SDYs density variable. As shown in Table 7, the estimated effects of the movement on the three cognitive ability outcomes remained statistically significant at the 1% level across all SDYs density specifications, though the economic magnitude of the coefficients varied slightly. These results underscore the robustness of our findings to alternative measurement strategies.

**Table 7:** Robustness check: changing the measure of exposure to SDYs.

**Variables**	**MMSE score**	**Cognitive decline**	**Cognitive impairment**
	**(1)**	**(2)**	**(3)**
**Panel A: the median density of SDYs across all counties within**			
**a city**			
local density of received SDYs × affect cohorts (1956–1969)	1.634^***^	−0.0872^***^	−0.124^***^
	(0.169)	(0.0136)	(0.0163)
Observations	14,671	9,636	14,671
*R* ^2^	0.109	0.035	0.069
**Panel B: the maximum density of SDYs across all counties**			
**within a city**			
local density of received SDYs × cohorts (1956–1969)	19.200^***^	−1.026^***^	−1.515^***^
	(5.488)	(0.247)	(0.482)
Observations	14,671	9,636	14,671
*R* ^2^	0.109	0.035	0.070
**Panel C: the minimum density of SDYs across all counties**			
**within a city**			
local density of received SDYs × cohorts (1956–1969)	92.850^***^	−3.836^***^	−7.715^***^
	(18.56)	(1.315)	(1.450)
Observations	14,671	9,636	14,671
*R* ^2^	0.109	0.033	0.070
Controls	Yes	Yes	Yes
Cohort FE	Yes	Yes	Yes
City FE	Yes	Yes	Yes
Year FE	Yes	Yes	Yes

Standard errors are clustered at the city level. ^***^, ^**^ and ^*^ denote significance at 1%, 5% and 10% level, respectively.

## 7 Conclusion

This study utilizes data from the CHARLS conducted between 2011 and 2018 to explore the impact of the send-down movement on the cognitive ability of rural older adults in China. The findings indicate that childhood exposure to SDYs significantly improved the cognitive health of rural older adults. Specifically, individuals who were exposed to SDYs exhibited notably higher MMSE scores than those who were not, experienced slower cognitive decline, and had a significantly reduced probability of developing cognitive impairments. These results remained robust after a series of rigorous checks, suggesting that the historical Send-down movement contributed to enhancing cognitive levels in rural areas, thereby promoting the overall health of the rural older adults.

Mechanism analysis further reveals that the impact of exposure to SDYs on cognitive ability was primarily mediated through the improvement of educational levels in rural areas. First, SDYs who served as primary school teachers directly enhanced the education levels of rural children by imparting new knowledge and culture. Second, their interactions with rural children broadened the children's horizons. As these children grew up, they were more likely to transition away from agricultural labor and engage in non-agricultural work. This shift was accompanied by an expansion of their social networks and an increase in social activities, both of which positively influenced cognitive development. Additionally, influenced by the one-child policy in urban areas, older adults in regions exposed to SDYs were more likely to have fewer children, which helped alleviate life pressures and created favorable conditions for cognitive enhancement.

Despite the strong evidence demonstrating the positive impact of exposure to SDYs on the cognitive ability of rural older adults, this study has some limitations. First, there were regional differences in the historical context and policy implementation of exposure to SDYs, which may have led to varying effects across different areas. Second, this study primarily focuses on the impact of exposure to SDYs on the cognitive ability of rural older adults and does not explore other potential effects of the policy, an area that warrants further investigation in future research.

Overall, this study highlights the profound influence of exposure to SDYs on the cognitive ability of rural older adults and elucidates the underlying mechanisms through four pathways: education improvement, non-agricultural job opportunities, social activities, and reduced family size. These findings provide an important perspective for understanding the impact of social change on individual cognitive ability and offer valuable policy insights for improving the cognitive health of rural older adults.

## Data Availability

The raw data supporting the conclusions of this article will be made available by the authors, without undue reservation.
